# Commentary: Potential Therapeutic Consequences of an Acid-Sensing Ion Channel 1a-Blocking Antibody

**DOI:** 10.3389/fphar.2019.00954

**Published:** 2019-08-30

**Authors:** Andrew Peterson, Qian Jiang, Xiang-Ping Chu

**Affiliations:** ^1^Department of Biomedical Sciences, School of Medicine, University of Missouri-Kansas City, Kansas City, MO, United States; ^2^Neuroscience Laboratory for Translational Medicine, School of Mental Health, Qiqihar Medical University, Qiqihar, China

**Keywords:** acid-sensing ion channels, stroke, ischemic brain injury, neuroprotection, monoclonal antibody

## Introduction

Strokes are the second leading cause of death in the world (Patel and McMullen, 2017). Of all strokes, ischemic strokes make up ∼87% of them, making safe and effective therapy necessary (Powers et al., 2018). Ischemic strokes have been known to decrease oxygen to brain, resulting in anaerobic glycolysis and lactic acid production, lowering the pH. One of the critical candidates for sensing acidosis is acid-sensing ion channels (ASICs) (Waldmann et al., 1997; Xiong et al., 2004; Yermolaieva et al., 2004). ASICs have been shown to be an important target for ischemia-induced neuronal damage due to their function in the pathological state of acidosis resulting from the ischemic stroke. ASIC1a, in particular, is known to have calcium permeability and has been shown to be the critical target of ischemia-induced brain damage (Xiong et al., 2004; Yermolaieva et al., 2004). Thus, ASIC1a seems to be more active during pathological conditions than physiological processes (Chu et al., 2014). In more recent years, the focus has been on potential therapeutic agents that can selectively block ASIC1a to ultimately decrease neuronal injury following ischemic stroke (Huang et al., 2015). Some of the potential agents include small molecule drugs, such as amiloride and flurbiprofen (Mishra et al., 2010; O’Bryant et al., 2014), and venom peptides, such as PcTx1 (Salinas et al., 2006) and Hi1a (Chassagnon et al., 2017; Ren et al., 2018). Amiloride and nonsteroidal anti-inflammatory drugs like flurbiprofen are nonspecific inhibitors of ASICs (O’Bryant et al., 2014). Previous studies have shown flurbiprofen to be efficacious only before an acute ischemic stroke, and it is not effective up to 4 h after a stroke (Mishra et al., 2010). While venom peptide PcTx1 has shown to be highly selective and efficacious at ASIC1a, venom peptides are biologically unstable in humans, making them inadequate for therapy (Qiang et al., 2018). Therefore, there is an urgent need for a therapy that is specific, effective, and safe while allowing the clinician >4.5 h of time window that tissue plasminogen activator (tPA) has after a stroke.

## ASC06-IgG1 Inhibits ASIC1a

A recent study was reported in the *Proceedings of the National Academy of Sciences* (Qiang et al., 2018). Qiang et al. discovered a novel therapy for reducing ischemia-induced neuronal death using a selective ASIC1a-blocking monoclonal antibody (mAb), ASC06-IgG1, in an experimental ischemic model. The researchers generated mAb specific to the ASIC1a and found that the ASC06-IgG1 showed potent, sustained, and highly selective inhibition on ASIC1a. In our previous study, PcTx1 has been identified as a potent and selective inhibitor of ASIC1a and demonstrated great neuroprotection in ischemic brain injury (Xiong et al., 2004). In the present study by Qiang et al., they found that PcTx1 showed nearly complete inhibition of human ASIC1a (hASIC1a) current in expressed cells; however, ASC06-IgG1 displayed sustained and dose-dependent inhibition of up to 80% of the pH 6.0-induced ASIC currents. Furthermore, ASC06-IgG induced sustained inhibition, which is not reversible after washout for 30 min, while PcTx1 could be fully washed within 5 min. Mechanically, PcTx1 reveals its activity on ASIC1a through regulating steady-state desensitization of ASICs; however, ASC06-IgG1 seemed to use a mechanism that is noncompetitive with proton concentration. ASC06-IgG1 showed a dose-dependent inhibition of calcium influx with an IC_50_ of ∼3 nM, while amiloride at a concentration of 30 µM showed only 21% inhibition. When assessing tolerance, ASC06-IgG1 displayed no major degradation or aggregation of the antibody, whereas PcTx1 showed some tolerance when used as an analgesic for pain (Mazzuca et al., 2007). While comparing the neuroprotective effects of ASC06-IgG1 to PcTx1, they found that it reduced infarct volume to a similar degree as PcTx1 in a mouse model of middle cerebral artery occlusion (Qiang et al., 2018).

## Discussion

Antibodies have been applied in neurodegenerative diseases (Katsinelos et al., 2019). This study sheds new light on ASC06-IgG1 as a novel mAb for translational stroke research. Monoclonal antibodies (mAbs) have been used for years in treating certain diseases, especially malignants cancers/tumors (Pento, 2017). Of the many current mAbs on the market, two of them include rituximab, which is an anti-CD20 antibody that was approved by the Food and Drug Administration for treatment of B-cell non-Hodgkin lymphoma, and trastuzumab, which is an anti-HER2 receptor antibody used in the treatment of HER2-overexpressing breast and gastric cancer (McLaughlin et al., 1998; Bang et al., 2010; Slamon et al., 2011). Because of the increase in successful treatment of systemic metastasis outside the blood–brain–barrier (BBB) by mAbs, there has been relative increases in metastasis of cancers to the central nervous system (CNS), highlighting the urgent need in treatment of metastasis of cancers by mAbs (Weidle et al., 2015). An important factor to consider with any mAb is the route of administration in order to allow the antibody to reach the target tissue in the highest concentrations. The BBB is a barrier to prevent certain substances and drugs from reaching the CNS from the blood stream. If an antibody is administered intrathecally but is unable to escape the CNS to the bloodstream, it could produce prolonged toxic side effects (Upadhyay, 2014). Qiang et al. injected the antibody intracerebroventricularly, giving it direct access to the CNS, which is a technique that has been used for certain antimicrobials in humans (Cook et al., 2009). Another possible route of administration that has been used with antibodies is the intrathecal route. Intranasal route has been used for the administration of venom peptides that inhibit the ASIC1a channel to mice (Chassagnon et al., 2017), but it is unlikely that that an antibody could be administered in this way due to size constrictions. However, recent study has shown that antibody-conjugated nanoparticles can be used to target encapsulated drugs, which can cross the BBB and reach the brain cells, and have proven to be very promising (Loureiro et al., 2014; Loureiro et al., 2017; Teleanu et al., 2019). Therefore, the delivery of the mAb ASC06-IgG1 by nanoparticles to the ischemic brain region and the time window for ASC06-IgG1 of over 3 h are in need for further study.

## Author Contributions

All authors listed have made substantial, direct and intellectual contribution to the work, and approved it for publication.

## Funding

This work was partly supported by grants from the Sarah Morrison Student Research Award of University of Missouri-Kansas City School of Medicine to AP, University of Missouri Research Board, Missouri, USA, the Qiqihar Medical University (QY2016ZD-01), Heilongjiang Province, China and American Heart Association (19AIREA34470007) to X-PC.

## Conflict of Interest Statement

The authors declare that the research was conducted in the absence of any commercial or financial relationships that could be construed as a potential conflict of interest.
